# Utility of Psychometric Hepatic Encephalopathy Score in Diagnosing Minimal Hepatic Encephalopathy Among Liver Cirrhosis Patients in Nigeria

**DOI:** 10.7759/cureus.94682

**Published:** 2025-10-15

**Authors:** Mansur F Mohammed

**Affiliations:** 1 Gastroenterology, University Hospitals of Derby and Burton NHS Trust, Burton, GBR

**Keywords:** hepatic encephalopathy, liver cirrhosis, minimal hepatic encephalopathy, nigeria, psychometric hepatic encephalopathy score

## Abstract

Introduction: Hepatic encephalopathy (HE) is a critical complication of liver cirrhosis, with minimal hepatic encephalopathy (MHE) representing a subtle neurocognitive disorder within its spectrum, characterized by cognitive and motor impairments undetectable by routine clinical examinations. The Psychometric Hepatic Encephalopathy Score (PHES), comprising five psychometric tests, is the gold standard for MHE diagnosis, enabling early intervention to prevent accidents, falls, progression to overt HE, and reduced quality of life. The study aimed to establish a local PHES reference value in Nigeria and evaluate its utility in diagnosing MHE among liver cirrhosis patients.

Methods: It is a cross-sectional study; 200 apparently healthy controls and 85 liver cirrhosis patients without overt HE underwent PHES testing, including Number Connection test-A (NCT-A), Number Connection test-B (NCT-B), Digit Symbol Test (DST), Serial Dotting Test (SDT), and Line Tracing Test (LTT).

Results: The mean age of the liver cirrhosis patients was 40.5 ± 11.8 years, with a male-to-female ratio of 2.4:1, comparable to the controls. MHE was diagnosed at PHES < -5, revealing a prevalence of 51.8%. Risk factors associated with MHE included serum albumin, prothrombin time, and Child-Pugh score (p < 0.05), with Child-Pugh score (OR 9.3) and prothrombin time (OR 19.5) as independent predictors. Subtest combinations (NCT-A + NCT-B, NCT-A + SDT) showed sensitivities of 84.1% and 86.4%, respectively, and 61% specificity, reducing test time by 52%.

Conclusion: The high MHE prevalence underscores the need for PHES validation across Nigeria to develop national diagnostic guidelines.

## Introduction

Hepatic encephalopathy (HE) is a severe complication of chronic liver disease (CLD), characterized by a spectrum of neuropsychiatric abnormalities, including cognitive, psychomotor, and motor coordination deficits [1,2]. Minimal hepatic encephalopathy (MHE), the mildest form, affects 10-84% of cirrhosis patients, with prevalence varying due to diverse diagnostic tools and cut-off points [3,4].

MHE, though subclinical, significantly impacts patients, increasing risks of road traffic accidents, falls, hospitalization, and progression to overt HE, thus reducing quality of life and increasing mortality [5-7].

The Psychometric Hepatic Encephalopathy Score (PHES), comprising five tests - Number Connection test-A (NCT-A), Number Connection test-B (NCT-B), Digit Symbol Test (DST), Serial Dotting Test (SDT), and Line Tracing Test (LTT) - is the internationally recommended gold standard for MHE diagnosis according to the Working Party at the 11th World Congresses of Gastroenterology, Vienna, 1998, and the International Society of Hepatic Encephalopathy and Nutrition Metabolism (ISHEN) guidelines due to the wide domains of encephalopathy it assesses, including motor speed, attention, visual perception, and memory, compared to other modalities like Critical Flicker Frequency and Repeatable Battery for the Asssessment of Nueropsychological Status (RBANS) [8-10].

Standardized in countries like Germany, Italy, and China, PHES lacks validation in Nigeria, necessitating research to determine local reference values due to educational and cultural differences, which impact number and English letter proficiencies despite education years [11-13]. Due to these cultural, learning, and educational differences, age and education influence PHES performance, requiring statistical adjustments to ensure diagnostic accuracy [10,14-16].

This study aimed to establish a PHES reference value for Kano city, determine MHE prevalence, evaluate subtest sensitivity and specificity, and identify associated risk factors among liver cirrhosis patients.

## Materials and methods

Study design

This cross-sectional observational study was conducted at Aminu Kano Teaching Hospital, Kano, Nigeria, with ethical clearance from the hospital’s ethics committee and informed consent from all participants.

Study population

In the treatment group, 85 known liver cirrhosis patients (aged 18-65 years, with at least primary education, no overt HE) were consecutively recruited from the Gastroenterology Clinic. Exclusion criteria included recent use of laxatives, psychoactive drugs, antibiotics, overt HE, neurologic/psychiatric disorders, or alcohol consumption (>30 g/day).

The control group consisted of 200 age- and sex-matched healthy adults attending routine check-ups at the General Outpatient Department were enrolled. Exclusion criteria included liver disease signs, obesity (BMI > 30), positive hepatitis B/C screening, or neurological/psychiatric disorders.

Sample size

Sample size was calculated using a 5.2% MHE prevalence from a European study [17], yielding 85 liver cirrhosis patients and 200 controls for establishing reference values at 99% confidence [18]. This study was used for sample size calculation, as there were no Nigerian studies using the PHES tool at the time of the study.

Materials and procedure

PHES tests (NCT-A, NCT-B, DST, SDT, LTT) were administered in a quiet, well-lit room between 9 AM and 2 PM by a trained interviewer who ensures the tests are completed as quickly as possible and errors are corrected by the subjects. Child-Pugh scores were calculated for all patients. Laboratory tests included albumin, prothrombin time, and hepatitis serology. Controls underwent ultrasound and hepatitis screening. All costs were covered by the researcher.

Data analysis

Data were analyzed using SPSS v20.0 (IBM Corp., Armonk, NY). PHES scores were calculated using a norm-based system, with MHE diagnosed at < -5 (mean - 2SD). Pearson’s correlation and multiple linear regression were used to correct for the effect of age and education. Associations were tested using chi-square and t-tests (p < 0.05). Cohen's d was used to calculate the effect size. Logistic regression identified independent MHE predictors. Sensitivity, specificity, and ROC analysis evaluated various PHES subtest combinations.

## Results

Control subjects

The control subjects (mean age 39.0 ± 11.0 years, 70.5% male) had the following mean PHES test times: NCT-A, 48.2 ± 21.4s; NCT-B, 102.7 ± 34.4s; DST, 33.1 ± 9.9s; SDT, 115.9 ± 30.2s; LTT, 205.8 ± 60.7s. The PHES cut-off for MHE was calculated as <-5, based on mean - 2SD.

Age and education years were found to significantly correlate with PHES scores (p < 0.001), but sex did not (p > 0.05). Multiple linear regression-derived predictive formulas for PHES tests, adjusting for confounders (Table 1). 

**Table 1:** Equations for Predicting PHES Tests Results Ffrom Age and Education Years in the Control Group PHES: Psychometric Hepatic Encephalopathy Score; NCT-A: Number Connection Test A; NCT-B: Number Connection Test B; DST: Digit Symbol Test; SDT: Serial Dotting Test; LTT: Line Tracing Test

PHES Test	Equation	SD
NCT-A	42.37 + (0.565 x age) – (1.454 x education years)	6.4
NCT-B	100 + (0.677 x age) – (2.212 x education years)	10.64
DST	35 – (0.187 x age) + (0.457 x education years)	3.16
SDT	100 + (0.716 x age) – (1.162 x education years)	9.61
LTT	187 + (1.023 x age) – (1.918 x education years)	19.93

Liver cirrhosis patients

Among the 85 liver cirrhosis patients had the mean age was 40.5 ± 11.8 years, 72.9% male, and 42.4% had at least secondary education. Hepatitis B was the predominant cause of liver cirrhosis, prevalent in 77.6%.

The mean Child-Pugh Score was 7.4 (SD 1.7, range 5-11). Around 62% of the patients were classified as either Child-Pugh Class B or C. 

The mean PHES was -6.6 ± 3.7 (range -12 to 3). Using PHES <-5 as an abnormal cut-off, MHE prevalence was determined as 51.8%. Significant risk factors for MHE were low albumin (p = 0.020), prolonged prothrombin time (t-test, p < 0.001), and higher Child-Pugh class (chi-square 13.87, df 2, p = 0.001) as shown in Table 2. Logistic regression confirmed Child-Pugh class (OR 9.4) and prothrombin time (OR 19.5) as independent predictors, as shown in Table 3.

**Table 2:** Factors Associated With Minimal Hepatic Encephalopathy *Statistically significant. MHE: minimal hepatic encephalopathy; DF: degree of freedom; HBsAg: hepatitis B surface antigen; ALT: alaninine aminotransferase; AST: aspartate aminotransferase; µmol/L: micromol per litre; S: seconds

Variables	MHE	No MHE	Test	Test value	P value	DF	Effect size
Age (Years)	39.6 ± 12.2	41.5 ± 11.3	T test	0.75	0.45	83	0.16
Sex (M/F)	32/12	30/11	X^2^	0.01	0.96	1	0.01
Residence (urban/rural)	25/19	26/15	X^2^	0.39	0.54	1	0.07
Ascites (present/absent)	31/13	28/13	X^2^	1.91	0.39	2	0.15
HBSAg (+ve/-ve)	34/10	32/9	X^2^	0.07	0.93	1	0.01
Child-Pugh class			X^2^	13.87	0.001*	2	0.4
Class A	9	23					
Class B	24	16					
Class C	11	2					
Albumin	32.1±9.2	36.4±7.6	T test	2.37	0.020*	83	0.52
Creatinine	84.4 ± 21.69	76.0 ± 20.2	T test	2.04	0.064	83	0.44
Prothrombin time (S)	21.6 ± 4.4	15.9 ± 3.4	T test	6.87	0.000*	83	1.49
Platelet count (x10^9^)	202.3 ± 107.5	192.2 ± 86.3	T test	0.47	0.64	83	0.1
Bilirubin (µmol/L)	26.3 ± 15.6	28.1 ± 37.2	T test	0.29	0.77	83	0.06
ALT (µmol/L)	30.0 ± 16.9	37.0 ± 67.1	T test	0.67	0.51	83	0.14
AST (µmol/L)	45.9 ± 31.4	56.5 ± 58.8	T test	1.05	0.29	83	0.23

**Table 3:** Logistic Regression of Significant Risk Factors of Minimal Hepatic Encephalopathy *Statistically significant, g/l: gram per litre

Variable	P value	Odds Ratio	95% Confidence Interval	
			Lower	Upper
Albumin (g/l)	0.181	1.063	0.972	1.163
Prothrombin time (s)	0.000*	19.513	4.368	87.169
Child-Pugh class	0.002*	9.37	2.643	14.056

Subtest combinations NCT-A + NCT-B and NCT-A + SDT showed sensitivities of 84.1% and 86.4%, respectively, and 61% specificity, with AUCs of 0.725 and 0.737, reducing test time by 52%.

## Discussion

This study established a PHES cut-off of < -5 for diagnosing MHE in Nigeria, aligning with findings from Indian studies [19,14] but differing from European and Chinese studies, which reported a cut-off of < -4 [20, 16]. This discrepancy underscores the necessity of local standardization, as educational levels, cultural factors, and literacy rates significantly influence PHES performance [9]. However, the effect of confounders such as education and age was removed using multiple linear regression formulas, and this limited their effect on the results to a large extent.

The high MHE prevalence of 51.8% in this study is comparable to reports from Egypt (50%) and China (49%) [21, 16], but notably higher than in Germany (25%) and Mexico (15%) [20, 22]. This variation may be attributed to the advanced liver disease in our cohort, with 47.1% of patients in Child-Pugh class B and 15.3% in class C, reflecting more severe hepatic dysfunction [23].

The significant association of MHE with Child-Pugh class, prolonged prothrombin time, and low serum albumin aligns with findings from Romanian and Indian studies [23, 14], suggesting that these parameters reflect underlying portal hypertension and impaired liver synthetic function, key contributors to MHE pathogenesis [24]. Logistic regression identified Child-Pugh class (OR 9.4) and prothrombin time (OR 19.5) as independent predictors, consistent with prior research linking disease severity to MHE risk [14]. The high odds ratio for prothrombin time may indicate cholestasis, a known factor in portal hypertension, exacerbating neurocognitive deficits [24].

The use of subtest combinations (NCT-A + NCT-B, NCT-A + SDT) achieved sensitivities of 84.1% and 86.4%, respectively, and 61% specificity, reducing administration time by 52%, which is particularly valuable in resource-limited settings [16]. These findings are consistent with a Chinese study reporting similar accuracy for NCT-A + NCT-B (AUC 0.7) [16]. The reduced test time enhances clinical feasibility, addressing the practical challenge of administering the full PHES battery, which takes approximately 25 minutes [8].

Limitations include the absence of prior Nigerian or African PHES studies at the time of study; hence, a European study was used to calculate the sample size of the cirrhotic cohort. It also limited direct comparisons. Additionally, the single-center design may not fully represent Nigeria’s diverse population, necessitating future multicenter validation studies. Future research should also explore the use of online calculators to simplify PHES scoring.

## Conclusions

The PHES is a reliable tool for MHE diagnosis in Nigeria, with a cut-off of < -5, giving a high prevalence of MHE. Child-Pugh class and prothrombin time are key predictors, and subtest combinations reduce testing time while maintaining accuracy. Validation studies across Nigeria are recommended to establish national MHE diagnostic guidelines, potentially integrating online tools for rapid PHES calculation.
